# Hydrothermally Grown MoS_2_ as an Efficient Electrode Material for the Fabrication of a Resorcinol Sensor

**DOI:** 10.3390/ma16031180

**Published:** 2023-01-30

**Authors:** Huda Alsaeedi, Ali Alsalme

**Affiliations:** Department of Chemistry, College of Science, King Saud University, Riyadh 11451, Saudi Arabia

**Keywords:** MoS_2_, resorcinol, electrochemical sensor, linear sweep voltammetry

## Abstract

Recently, the active surface modification of glassy carbon electrodes (GCE) has received much attention for the development of electrochemical sensors. Nanomaterials are widely explored as surface-modifying materials. Herein, we have reported the hydrothermal synthesis of molybdenum disulfide (MoS_2_) and its electro-catalytic properties for the fabrication of a resorcinol sensor. Structural properties such as surface morphology of the prepared MoS_2_ was investigated by scanning electron microscopy and phase purity was examined by employing the powder X-ray diffraction technique. The presence of Mo and S elements in the obtained MoS_2_ was confirmed by energy-dispersive X-ray spectroscopy. Finally, the active surface of the glassy carbon electrode was modified with MoS_2_. This MoS_2_-modified glassy carbon electrode (MGC) was explored as a potential candidate for the determination of resorcinol. The fabricated MGC showed a good sensitivity of 0.79 µA/µMcm^2^ and a detection limit of 1.13 µM for the determination of resorcinol. This fabricated MGC also demonstrated good selectivity, and stability towards the detection of resorcinol.

## 1. Introduction

Environmental pollution is one of the major concerns for today’s world [1,2,3,4]. Various toxic compounds drain out from industry. These toxic compounds may have negative impacts on humans and the environment [3]. Particularly, resorcinol (RS, 1,2-benzenediol) is the derivative of phenolic compounds and has been widely used in various applications such as the dye food and pharmaceutical industry [5,6]. RS has also been explored in other areas including hair dye, bleaches, and skin peels, but has high toxicity and stability [7]. It is believed that decomposition of RS is very tough in the ecological environment. RS can be easily spread in the natural environment and it can influence human health [8,9,10]. RS may cause various health-related issues such as scalp and kidney damage, cyanopathy, convulsion, liver infections, and catarrh dermatitis [9,10,11]. Additionally, accidental intake of RS directly causes the cyanosis, respiratory failure, central nervous system, seizures, unconsciousness, and drowsiness [11,12,13]. Thus, it is necessary to develop methods for the detection of RS.

In recent years, conventional techniques, including high-performance liquid chromatography, flow injection, capillary electrophoresis, chemiluminescence, spectrophotometry, quartz crystal microbalance, and surface plasmon resonance, have been widely used for the detection of RS [14,15,16,17,18]. Although conventional methods can be used for the determination of RS, they are time consuming, need more sophisticated equipment, are expensive and more space is required to install the instruments [19,20]. Thus, it is really very important to find other fast and cost-effective strategies to fabricate highly efficient sensing devices for RS determination.

Previous years have witnessed the rapid surge in the construction of electrochemistry-based sensors [21,22,23]. A large number of electrochemical sensors, such as ascorbic acid, urea, nitro group-contacting phenols, catechol, hydrogen peroxide, hydrazine, etc., have been reported using electrochemical methods [22,23,24,25]. In addition, electrochemical methods have many advantageous over conventional methods in terms of sensitivity, time consumption, simplicity, selectivity, cost-effectiveness, and portability [26]. The sensing ability/performance of electrochemical sensors is greatly affected by the physiochemical properties of nanomaterials.

Recently, two-dimensional (2D) materials, such as carbon materials, phosphides, halides, oxyhalide, chalcogenides, nitrides and layered silicate materials, have received great interest because of their excellent optoelectronic and physiochemical features [27]. Particularly, transition metal dicalcogenides have emerged as 2D-layered materials with ultrathin [28]. The number of layers, degree of crystallinity, and stacking sequences in their crystal structure largely influence the properties of transition metal dicalcogenides [29]. Molybdenum disulfide (MoS_2_) is a transition metal dicalcogenide which has drawn immense attention from the scientific community due to its excellent electronic properties, extraordinary crystal structure and optical properties [30]. The layers of Mo atoms are organized in a hexagonal array which is sandwiched between S layers [30]. The Mo-S is strongly held with covalent bonds while van Der Waal interactions exist between the S layers [30,31]. Presently, MoS_2_ has a unique place as a graphene analog. The MoS_2_-modified electrodes hold great promise for the fabrication of the next generation of electrochemical-sensing devices.

In this study, our group prepared MoS_2_ using a hydrothermal method and explored MoS_2_ as a sensing material for the development of an RS sensor. According to our literature survey, we did not found any previous report on the use of MoS_2_ as an electrode material for the detection of RS. This is the first research works which demonstrate the electro-catalytic role of MoS_2_ for the detection of RS. The MoS_2_-modified electrode exhibited excellent performance towards the detection of RS.

## 2. Experimental Section

### Chemical

Sodium molybdate (Na_2_MoO_4_·2H_2_O; Sigma, St. Louis, MO, USA), thiourea (NH_2_CSNH_2_; Merck, Rahway, NJ, USA), phosphate-buffered solutions (PBS; SRL), nitrophenol (Sigma), glucose (Merck), urea (Alfa-Aesar, Haverhill, MA, USA), uric acid (Merck), catechol (TCI), hydrazine (Sigma), hydrogen peroxide (Merck), ascorbic acid (Merck), and dopamine (Alfa-Aesar) were used as received. No further purification was carried out and the purchased chemicals and reagents were used as received. Synthesis of MoS_2_ and other experimental details are provided in supporting information.

## 3. Results and Discussion

### 3.1. MoS_2_ Characterization

**Figure 1:**
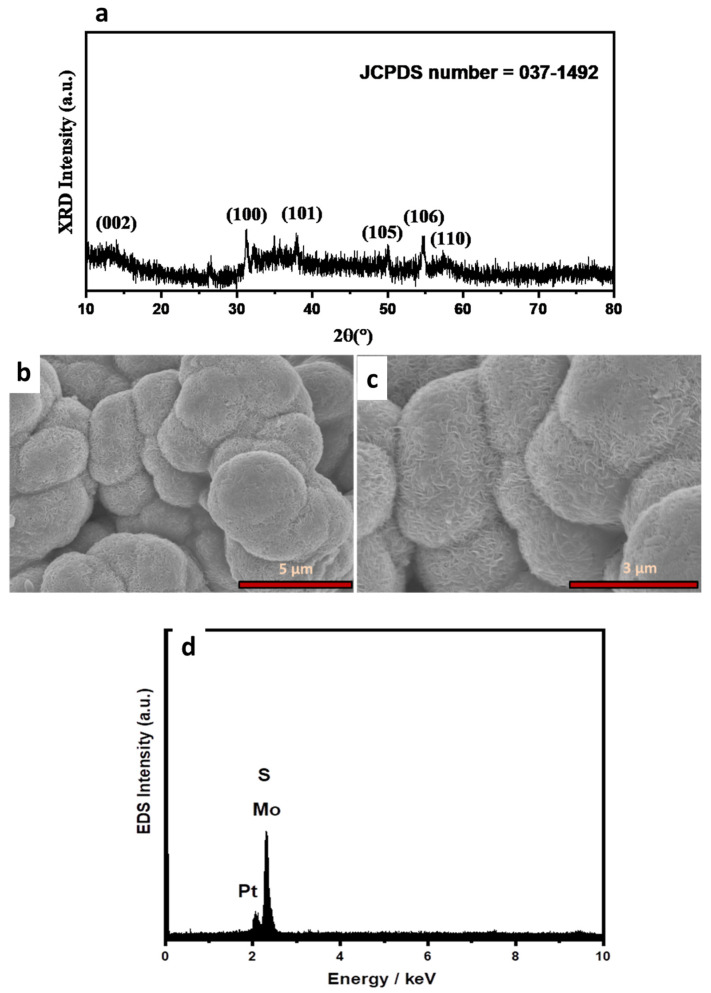
(**a**) PXRD and SEM images (**b**,**c**) and EDX spectrum (**d**) of the prepared MoS_2_.

The PXRD pattern for the synthesized MoS_2_ is shown in Figure 1a. The PXRD pattern of MoS_2_ shows the appearance of seven diffraction peaks. These diffraction peaks in the PXRD pattern of the MoS_2_ can be assigned to the (002), (100), (101), (105), (106), and (110) diffraction planes. These diffraction planes were in agreement with previous JCPDS card number 037-1492. Therefore, it can be said that MoS_2_ was obtained. Moreover, the absence of any other diffraction peak for impurities indicated the formation of MoS_2_ with good phase purity. The top view morphological characteristics of the obtained MoS_2_ were also studies using FE-SEM. The obtained FESEM images of the prepared MoS_2_ are shown in Figure 1b,c. The FE-SEM results clearly indicated the formation of hierarchical sheets like MoS_2_.

Furthermore, elemental analysis of the prepared MoS_2_ was also checked using EDS. The EDS spectrum of the prepared MoS_2_ is shown in Figure 1d. The EDS spectrum clearly indicated the presence of Mo and S corresponding to MoS_2_. Therefore, it was understood that MoS_2_ was successfully obtained (Figure 1d).

**Figure 2:**
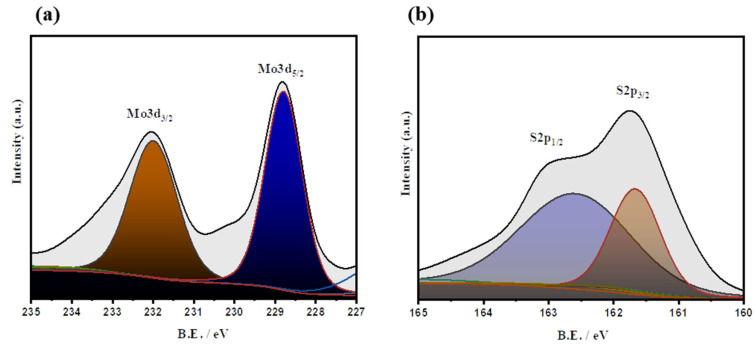
Mo3d (**a**) and S2p (**b**) XPS spectrum of the prepared MoS_2_.

XPS studies were also performed to further characterize the oxidation states of the elements present in the prepared MoS_2_. The Mo3d spectrum of MoS_2_ has been presented in Figure 2a. The Mo3d spectrum of MoS_2_ demonstrated two binding energy of 231.99 and 228.7 eV which may be ascribed to the presence of Mo3d_3/2_ and Mo3d_5/2_, respectively (Figure 2a) [32]. On other side, the S2p spectrum of MoS_2_ is shown in Figure 2b. Two binding energy of 162.62 and 161.66 eV were observed which can be assigned to the S2p_1/2_ and S2p_3/2_, respectively (Figure 2b) [32].

### 3.2. Electrochemical Sensing Properties

In the first stage, electrochemical impedance spectroscopy was used for investigating the electro-catalytic and charge-transfer properties of GC and MGC. The Nyquist plots of the GC and MGC were obtained in 0.1 M PBS containing 5 mM Fe(CN)_6_^3–/4–^(Amplitude: 5 mV, Frequency: 0.1 Hz to 100 kHz). The obtained results are shown in Figure S1. The GC exhibited the presence of a high-charge-transfer resistance whereas a low-charge-transfer resistance was observed for the MGC (Figure S1). This revealed that the MGC possessed good electro-catalytic properties and could be explored for electrochemical applications. The GC electrode was employed as a working electrode and its electrochemical performance and activity was studies in 50 µM RS in 0.1 M PBS of pH 7.0 (applied scan rate = 50 mV/s). In this work, we used PBS of pH 7.0 (0.1 M) for all electrochemical CV and LSV investigations. The GC electrode showed a very poor oxidation peak with a current response of ~2.14 µA. This is due to the poor active surface or electro-catalytic behavior of the non-modified GC electrode. The CV response of the MGC was further examined under a similar environment and conditions.

**Figure 3:**
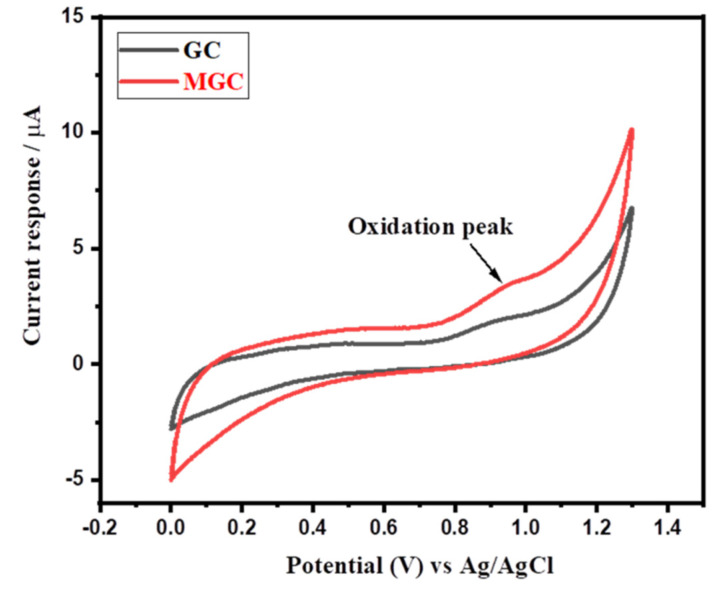
CV response of the GC and MGC for 50 µM RS at a scan rate of 50 mV/s.

**Figure 4:**
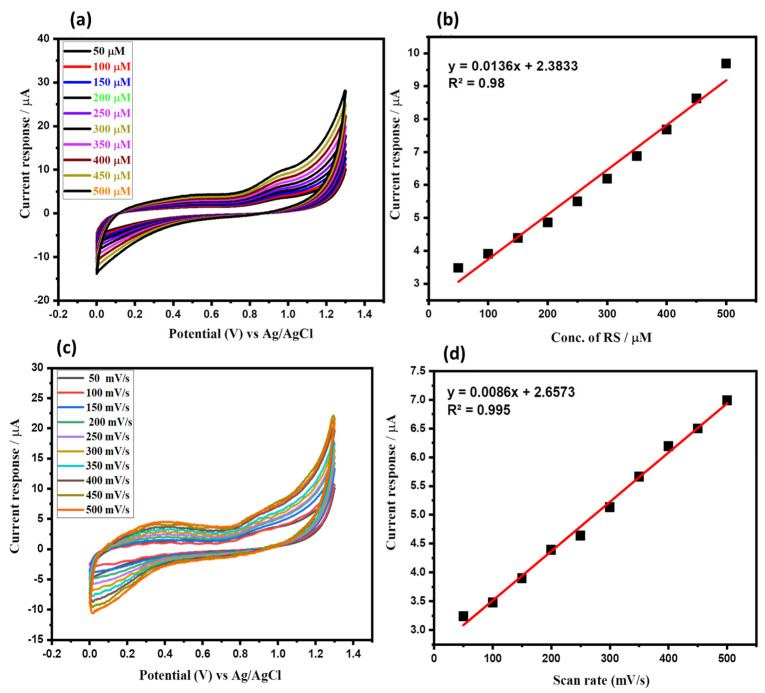
(**a**) CV responses and calibration plot (**b**) of the MGC for 50–500 µM RS at a scan rate of 50 mV/s. (**c**) CV responses and calibration plot (**d**) of the MGC for 50 µM RS at a scan rate of 50–500 mV/s.

The obtained CV response of the MGC for 50 µM RS is shown in Figure 3. In contrast, the MGC electrode exhibited an improved electro-catalytic current of 3.71 µA (Figure 3). This revealed that the MGC is superior over GC because of the presence of MoS_2_ on the surface of the GC. Thus, an enhanced electro-catalytic current response was observed for the MGC compared to the GC electrode under similar conditions (Figure 3) which prompted us to explore the MGC for further CV studies, such as the effect of various concentrations of RS and different applied potential scan rates. The above-obtained current response of ~2.14 µA for the GC and 3.71 µA for the MGC were the best current responses obtained. The GC and MGC demonstrated average current responses of 1.94 µA and 3.69 µA with relative standard deviations of 3.27 and 2.18, respectively. Therefore, the CV response of the MGC was also studied in different concentrations of RS under a fixed applied potential scan rate of 50 mV/s. The CV responses of the MGC in different concentrations of 50–500 µM are shown in Figure 4a. From the obtained CV responses, it can be seen that the electro-catalytic current response increased with the increasing concentration of RS. This indicated that the MGC had good electro-catalytic features for the electro-oxidation of a high concentration of RS via the CV method (Figure 4a). The calibrated plot (Figure 4b) also suggests that the current response is directly proportional to the concentration of RS and it increases linearly with an increasing concentration of RS.

Applied scan rate always influences the electro-catalytic behavior and properties of electrochemical sensors. The effect of various applied scan rates was also investigated on the electrochemical features of the MGC for the electro-oxidation of RS. The CV responses of the MGC were obtained for 50 µM RS at varied scan rates (50–500 mV/s). The CV responses of the MGC for 50 µM RS under various applied scan rates are shown in Figure 4c. The obtained results indicated that the current response of the MGC for 50 µM RS increased when changing the scan rate from 50 mV/s to 500 mV/s. This increasing current response for 50 µM RS was found to be linear as we can see that the current response increased with respect to the applied scan rate in Figure 4d. The obtained results indicated that 50 mV/s as a suitable scan rate for the detection of RS with a relatively visible oxidation peak.

Although CV studies showed decent results for the electrochemical oxidation of RS, the oxidation peak was not clearly observed. Therefore, we used the LSV technique for further electrochemical detection studies.

**Figure 5:**
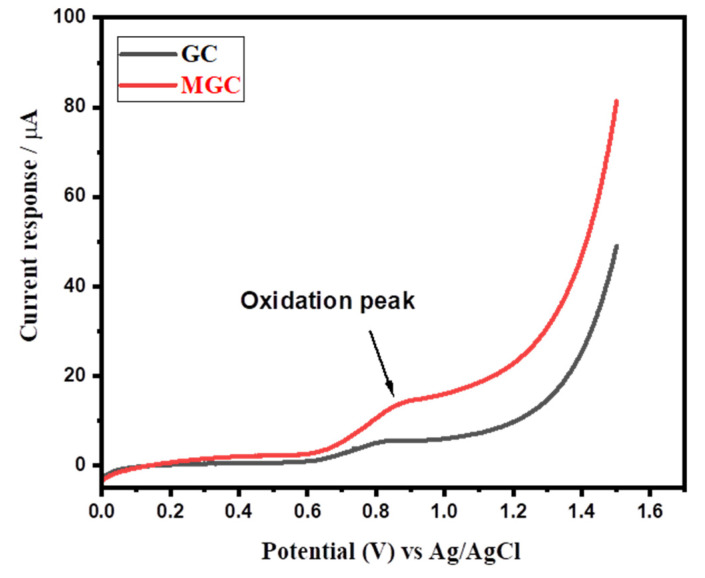
LSV responses of the GC and MGC for 50 µM RS at a scan rate of 50 mV/s.

**Figure 6:**
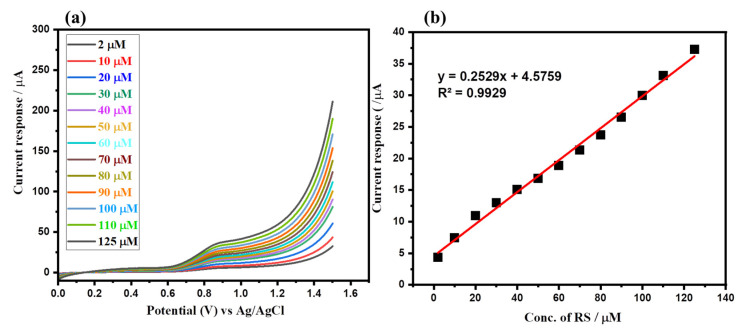
LSV responses (**a**) and calibration plot (**b**) of the MGC for 2–125 µM RS at a scan rate of 50 mV/s.

The LSV responses of the GC and MGC for 50 µM RS were recorded at an applied scan rate of 50 mV/s. The LSV responses of the GC and MGC for 50 µM RS are shown in Figure 5. The non-modified GC electrode showed a lower electro-catalytic response of 7.09 µA for the electro-oxidation of 50 µM RS. On contrary, the MGC electrode showed an improved electro-catalytic current response of 15.07 µM (Figure 5). This revealed that the MGC is highly electro-catalytic in nature for the electro-oxidation of RS which is due to the presence of MoS_2_. The LSV responses showed improved results for the electro-oxidation of RS compared to the CV. Thus, we further studied the influence of various concentrations of RS on the electro-catalytic current response of the MGC. The responses of the MGC were recorded in different concentrations (2–125 µM) of RS at an applied potential scan rate of 50 mV/s. The obtained LSV responses of the MGC in different concentrations of RS from 2 to 125 µM are shown in Figure 6a. According to Figure 6a, LSV responses suggested that the electro-catalytic current response increased with an increase in the concentration of RS. This revealed that the MGC had decent electro-catalytic properties towards the electro-oxidation of RS via the LSV method (Figure 6a). The calibrated plot also indicated that the electro-oxidation current response was directly proportional to the concentration of RS and increased linearly with an increase in the concentration of RS (Figure 6b).

A large number of electro-active compounds, such as nitro-phenol, glucose, urea, uric acid, catechol, hydrazine, hydrogen peroxide, ascorbic acid, dopamine, etc., are available which can affect the sensing behavior of the MGC for the determination of RS. It is necessary to study the anti-interfering nature of the constructed MGC. Thus, our research group studied the selective nature of the MGC in the presence of various electro-active compounds, such as nitro-phenol, glucose, urea, uric acid, catechol, hydrazine, hydrogen peroxide, ascorbic acid, and dopamine.

**Figure 7:**
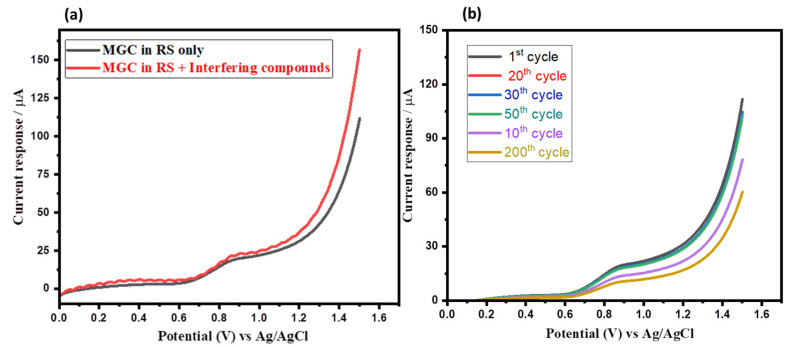
(**a**) LSV response of the MGC for 50 µM RS and 50 µM RS + 250 µM interfering compounds (nitrophenol, glucose, urea, uric acid, catechol, hydrazine, hydrogen peroxide, ascorbic acid, and dopamine) at a scan rate of 50 mV/s. (**b**) LSV responses (1st, 20th, 30th, 50th, 100th and 200th cycle) of the MGC for 50 µM RS at a scan rate of 50 mV/s.

The LSV response of the MGC was obtained for 50 µM RS at a scan rate of 50 mV/s. The obtained LSV response of the MGC for 50 µM is presented in Figure 7a. Further, the LSV response of the MGC for 50 µM RS + 250 µM interfering compounds (nitrophenol, glucose, urea, uric acid, catechol, hydrazine, hydrogen peroxide, ascorbic acid, and dopamine) was also recorded. The observations did not show any significant variation in the electro-catalytic current response. Therefore, it revealed the presence of a good anti-interfering nature of the MGC for RS determination. In previous years, various electrochemical sensors have been reported which studied the cyclic stability and repeatability. In our present work, we have also studied the cyclic stability and repeatability of the MGC for 50 µM RS at a scan rate of 50 mV/s.

**Scheme 1:**
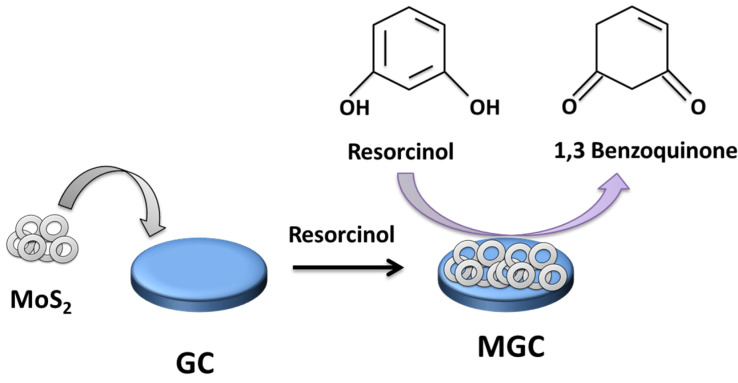
Schematic representation of the fabrication of the MGC.

The LSV responses of the MGC for 50 µM RS were recorded up to 50 cycles. The LSV response for the 1st, 20th, 30th, 50th, 100th and 200th cycle of the MGC are shown in Figure 7b. The obtained LSV responses show good cyclic stability and cyclic repeatability after 50 cycles. However, the MGC also retained more than 50% stability after 200 cycles. This stability may be attributed to the excellent 2D-layered structure of MoS_2_. The electro-oxidation process for RS sensing is described in Scheme 1. During the electrochemical-sensing process, 1, 3 benzoquinone formed in the electro-oxidation of RS, as shown in Scheme 1.

The electrochemical limit of detection (LoD) and sensitivity of the MGC for RS determination was calculated according to the equations listed below:

LoD = 3 × σ/S(1)

Sensitivity = S/A_e_ (2)

(σ = standard deviation, S = slope, and A_e_ = area of GC).

The MGC showed a reasonably good LoD of 0.13 µM and a sensitivity of 0.79 µA/µMcm^2^ which are summarized in Table 1 along with previously reported sensors [33,34,35,36,37,38,39,40,41,42].

According to previous studies, Huang et al. [32] synthesized novel composites of WS_2_ and graphene (WS_2_/Gr) via the L-cysteine-assisted solution phase approach. Further, authors have modified GCEs with WS_2_/Gr as an RS-sensing material and the CV technique was adopted for sensing analysis. The WS_2_/Gr/GCE showed a low LoD of 0.1 µM with a good dynamic linear range. Li et al. [33] also investigated the RS-sensing property of cerium phosphate nanotubes (CePO_4_). The CePO_4_ was deposited on the GCE and the electrochemical-sensing behavior of this modified electrode (CePO_4_/GCE) was studied. An LoD of 0.14 µM was reported for RS detection using the CePO_4_/GCE. Chetankumar et al. [34] prepared magnesium oxide (MgO) for the fabrication of an RS sensor. The MgO pre-treated carbon-paste electrode (MgO/MPCPE) was prepared and its electrochemical-sensing properties towards the detection of RS were studied by using a voltammetric approach. This MgO/MPCPE-based RS sensor exhibited an LoD of 0.25 µM [34]. Furthermore, Chen et al. [35] also investigated the sensing properties of a prepared Au-Pd nano-flower (NF)/rGO material. The GCE was fabricated with Au-Pd NF/rGO as an RS-sensing material. The authors found that the Au-Pd NF/rGO/GCE was highly sensitive towards the detection of RS and an excellent LoD of 0.7 µM was obtained [35]. Buleandra et al. [36] also fabricated an RS sensor by exploring the pencil graphite electrode (PGE). The PGE was electrochemically treated with cobalt-phthalocyanine (CoPC). This fabricated electrode (CoPC/PGE) exhibited an LoD of 0.72 µM by employing the DPV method [36]. Ershadifar et al. [37] also developed an RS sensor using the gold (Au) decoration approach. The surface of the GCE was fabricated with the prepared MWCNTs-Au on the GCE. This fabricated RS sensor (MWCNTs-Au/GCE) revealed that MWCNTs-Au possesses a good sensing nature and an LoD of 0.8 µM was obtained.

Ge et al. [38] prepared few-layered graphene (Gr) using the chemical vapor deposition technique. The few-layered Gr was deposited on a Ta thin substrate. Further, a ZnO/Gr composite was obtained on a Ta substrate and this fabricated ZnO/Gr/Ta electrode was adopted as an RS sensor. The electrochemical study showed that an LoD of 1 µM can be obtained for RS using a ZnO/Gr/Ta electrode. The electrochemical-sensing property of multi-walled carbon nanotubes (MWCNTs) for RS detection was studied by Golestaneh and co-workers [39]. The active area of the non-modified GCE was fabricated with MWCNTs and the sensing behavior of the MWCNTs/GCE towards RS determination was examined. The authors achieved an LoD of 1.1 µM for RS detection using the MWCNTs/GCE as a working electrode. Metal selenides, such as cobalt iron selenide (CoFe_2_Se_4_), have been widely used in various electrochemical-sensing applications but they have poor active sites and low conductivity. Thus, the active sites and conductivity of a three dimensional (3D) CoFe_2_Se_4_ were enhanced by introducing porous carbon nanofibers (3D-CoFe_2_Se_4_/PCF) using an electro-spinning method [40]. This obtained 3D-CoFe_2_Se_4_/PCF was explored as an RS sensor. The electrochemical sensing results showed that a reasonable LoD of 1.36 µM could be obtained. In another report by Esteban and co-workers, an RS sensor was also reported using unique strategies [41]. The authors investigated the electrochemical-sensing ability of the graphene screen-printed electrode (GSPE) by using a differential pulse voltammetry (DPV) method. The GSPE was further adopted as an RS sensor and the authors achieved a good LoD of 2.4 µM for RS determination. This revealed that the LoD can be affected by the type of electrode materials present on the surface of the GCE. Porous materials possess good electrochemical properties and provide better electron transportation during electrochemical-sensing processes. Similarly, Zhang et al. [42] obtained porous rGO and explored its potential role in the determination of RS via voltammetric methods. This work reported an LoD of 2.62 µM for the sensing of RS using a porous rGO-modified electrode. Layered materials, such as tungsten disulfide (WS_2_), have been widely explored in electrochemical-sensing applications because of the presence of strong bonding between the layers and weak interactions between interlayers. The surface morphological property of the sensing materials may play a vital role in achieving improved sensing parameters. Zinc oxide (ZnO) has excellent electro-catalytic activities and has been widely used in the sensing of various analytes. Ameen et al. [43] adopted a low-temperature hydrothermal approach for the preparation of ZnO. Cabbage-like ZnO nanostructure nanosheets (C-ZnO NSs) were obtained by Ameen et al. [43]. Further, an RS chemical sensor was developed. This developed RS sensor exhibited an LoD of 5.89 µM.

In the present study, we explored the potential role of MGC for the sensing of RS and obtained results demonstrating a reasonable LoD of 0.13 µM. Finally, it can be said that MGC possesses a good selective nature for the sensing of RS including a reasonable LoD, cyclic stability and repeatability. The obtained results in this study are comparable with previous studies as shown in Table 1.

## 4. Conclusions

In this study, a hydrothermal synthetic approach was utilized for the synthesis of MoS_2_. The synthesized MoS_2_ was characterized by various techniques, namely PXRD, SEM, EDX, and XPS. Subsequently, a resorcinol (RS) sensor was developed by modifying the surface of a glassy carbon (GC) electrode. MoS_2_ was used as an electro-catalyst for the fabrication of an RS sensor. Two voltammetric techniques (cyclic voltammetry and linear sweep voltammetry) were adopted to check the potential of the MGC towards the detection of RS. The MGC demonstrated excellent electro-catalytic properties and sensing behavior for the determination of RS. The MGC also showed a good detection limit, sensitivity, repeatability and stability.

## Figures and Tables

**Table 1:** Comparison of the sensitivity and LoD of the MGC against published literature [32,33,34,35,36,37,38,39,40,41,42,43].

Sensing Material	Detection Limit (µM)	Sensitivity (µA/µMcm^2^)	References
WS_2_-Gr/GCE	0.1	-	[32]
CePO_4_/GCE	0.14	-	[33]
MgO/MPCPE	0.25	-	[34]
Au-Pd NF/rGO/GCE	0.7	-	[35]
CoPC/PGE	0.72	-	[36]
MWCNTs-Au/GCE	0.8	-	[37]
ZnO/Gr/GCE	1	-	[38]
MWCNTs/GCE	1.1	-	[39]
3D-CoFeSe_4_-PCF/GCE	1.36	-	[40]
GSPE	2.4	-	[41]
Porous rGO/GCE	2.62	-	[42]
C-ZnO NSs	5.89	1.98	[43]
MGC	0.13	0.79	Present work

## Data Availability

Not applicable.

## Supplementary Materials

The following supporting information can be downloaded at: https://www.mdpi.com/article/10.3390/ma16031180/s1, Figure S1: Nyquist curve of GC and MGC in 0.1 M PBS containing 5 mM Fe(CN)_6_^3–/4–^. Amplitude: 5 mV, Frequency: 0.1 Hz to 100 kHz.
