# Supplementary material for: Hydrothermally Grown MoS2 as an Efficient Electrode Material for the Fabrication of a Resorcinol Sensor
Source: Materials (Basel). 2023 Jan 30;16(3):1180. doi: 10.3390/ma16031180 (PMC9920819; doi:10.3390/ma16031180)
Supplement: Supplementary file 1 [file materials-16-01180-s001.zip › materials-2110442-supplementary.pdf]

Hydrothermally grown MoS_2 as efficient electrode material for the fabrication of resorcinol sensor

Materials and methods

Synthesis of MoS_2

MoS_2 was prepared using hydrothermal method according to reported elsewhere [31]. In brief, 500 mg of $Na_2MoO_4.2H_2O$ was dissolved in 40 mL of deionized water. Further, 600 mg of NH_2CSNH_2 was added in the above solution. This reaction solution was stirred for half an hour at room temperature and transferred in to the steel autoclave and heated at 200 °C for 24 h under vacuum furnace. The as-obtained product was washed with ethanol and deionized water and dried at 70 °C overnight in vacuum oven.

Materials characterization

In this study, Rigaku (Japan), RINT 2500 V powder X-ray diffractometer with Cu Ka irradiation (λ=1.5406 Å) was used to obtain the powder X-ray diffractogram (PXRD) of the prepared MoS_2. The scanning electron microscopic (SEM) images of the MoS_2 were obtained on field emission scanning electron microscope (FESEM) Oxford instrument (Supra 55 Zeiss). The Energy dispersive X-ray spectroscopic (EDS) data of MoS_2 was collected using Oxford Instruments X-max, Aztec spectroscope. The X-ray photoelectron spectroscopic (XPS) data of the prepared MoS_2 was obtained on PHI 5000 VersaProbe III. Electrochemical investigations were performed on CH Instrument which was connected with computer. Three-electrode assembly was used for sensing investigations. GC was used as working, Ag/AgCl as reference and Pt-wire as counter electrode.

Modification of GC

In the first stage, sensing material (MoS_2) was dispersed ultrasonically in deionized (DI) water (2.5 mg MoS_2 in 1 mL DI water). In another step, cleaned GC (3 mm diameter; alumina slurry was used for cleaning) was modified with the prepared MoS_2 dispersion (9 ±0.2 μL) via drop-casting technique. Further, 4 μL nafion (5 wt%; Sigma) was coated on to the MGC to improve the adhesiveness of MoS_2 on GC. This modified electrode (MGC) has been employed as RS sensing material. The PBS of pH 7.0 (0.1 M) was used for all the electrochemical investigations. The surface modification or working mechanism of the MGC towards RS sensing can be seen in Figure S1.

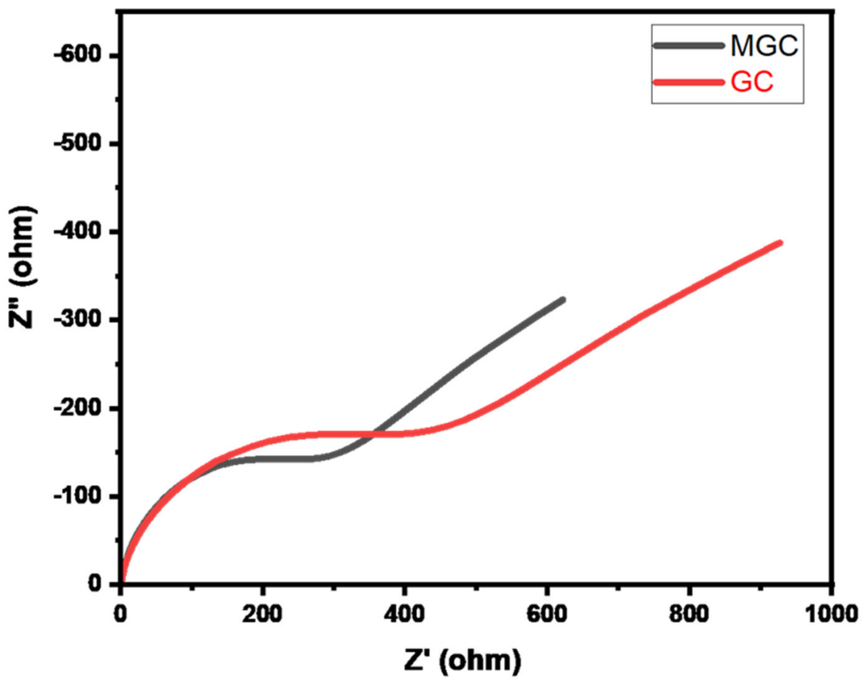

Figure S1. Nyquist curve of GC and MGC in 0.1 M PBS containing 5 mM $Fe(CN)_6^{3-/4-}$.

Amplitude: 5 mV, Frequency: 0.1 Hz to 100 kHz
